# The Influence Between *C-C Chemokine Receptor 5 *Genetic Polymorphisms and the Type-1 Human Immunodeficiency Virus: A 20-Year Review

**DOI:** 10.1089/aid.2022.0111

**Published:** 2023-01-11

**Authors:** Davi Silva Santana, Marcos Jessé Abrahão Silva, Ana Beatriz Rocha de Marin, Vanessa Ladyanne da Silva Costa, Gabriel Silas Marinho Sousa, Juliana Gonçalves de Sousa, Dihago Cardoso Silva, Eliete Costa da Cruz, Luana Nepomuceno Gondim Costa Lima

**Affiliations:** ^1^ Institute of Health Sciences (ICS), Federal University of Pará (UFPA), Belém, Brazil.; ^2^ Bacteriology and Mycology Section, Evandro Chagas Institute (IEC), Ananindeua, Brazil.; ^3^ Faculty of Physiotherapy, Maurício de Nassau College (UNINASSAU), Belém, Brazil.

**Keywords:** CCR5 receptor, genetic polymorphisms, HIV, cytokines

## Abstract

Acquired immune deficiency syndrome (AIDS) is an infectious disease caused by the types 1 and 2 human immunodeficiency virus (HIV-1 and HIV-2). Clinical outcomes in patients are highly varied and delineated by complex interactions between virus, host, and environment, such as with help of co-receptors, for example, the C-C chemokine receptor 5 (CCR5). This work aimed to describe the scientific evidence relating the influence of *CCR5* polymorphisms in association studies for HIV-1 disease susceptibility, severity, and transmissibility. This is a systematic review of the literature on single nucleotide polymorphisms (SNPs) and the deletion [Insertion and Deletion (Indel)] Δ32 of *CCR5*. The search for articles was based on the ScienceDirect, PubMed, and Coordination for the Improvement of Higher Education Personnel (CAPES) databases for the period between 2001 and 2021. The final sample consisted of 32 articles. ^†^SNP rs1799987 is one of the genetic polymorphisms most associated with the criteria of susceptibility and severity of HIV-1, having distinct consequences in genotypic, allelic, and clinical analysis in the variability of investigated populations. As for the transmission character of the disease, the G mutant allele of rs1799987 corresponds to the highest positive association. ^‡^Furthermore, the results on Indel Δ32 corroborate the absence and rarity of this variant in some populations. Finally, mitigating the severity of cases, SNPs rs1799988 and rs1800023 obtained significant attribution in individuals in the studied populations. It is shown that the reported polymorphisms express significant influences for the evaluation of diagnostic, therapeutic, and prophylactic measures for HIV-1 having fundamental particularities in the molecular, genetic, and transcriptional aspects of *CCR5*.

## Introduction

Acquired immune deficiency syndrome (AIDS) is a pathology caused by the human immunodeficiency virus (HIV) capable of weakening the immune system, mainly by affecting the CD4^+^ T lymphocytic cells. In terms of its morphological classification, the virus is characterized as a retrovirus from the *Retroviridae* family and subfamily *Lentiviridae*. It has structural and functional proteins in its structure, presents an encoded RNA genome protected by the viral envelope, which is constituted by a lipid bilayer and contains a complex protein called env, constituted in its external layer by glycoproteins gp41, transmembrane, and gp120.^1^ This virus shows a great genetic variability, besides having high mutation and recombination rates.^2^ This rapid evolutionary process has led to several HIV subtypes that are heterogeneously distributed globally.^3^ According to its phylogenetic classification, the virus can be subdivided into two types: type 1 HIV (HIV-1) and type 2 HIV (HIV-2). Based on this differentiation, this study will focus particularly on HIV-1.

HIV-1 is classified in four distinct groups, being represented by: M, N, O and P.^3^ Among these, the M group has the highest genetic prevalence among individuals and, therefore, the greatest worldwide dissemination, which has made it the main source of the global HIV pandemic and has a genetic variability configured by nine distinct strands with ^†^different strains: A, B, C, D, F, G, H, J, and K.^4^

Chemokines are cytokines that act on cell migration and activation in homeostasis and inflammatory conditions, in addition to regulating the traffic of immune cells from the bone marrow to the bloodstream and, subsequently, from the blood and lymph to the lymphoid and inflamed tissues; this mechanism occurs both in physiological situations and in autoimmune diseases.^5^ Thus, chemokine receptors have seven transmembrane compartments (7TMs) and belong to the group of G protein-coupled receptors (GPCRs), playing a key role in the ability of viral infection in specific cells.^6^ In this sense, the process of HIV infection of the host cell involves the interaction between the viral glycoprotein gp120 and molecules on the cell surface of the host cell. After using CD4 as the primary receptor, HIV interacts with the C-C chemokine receptor 5 (CCR5) or C-X-C chemokine receptor type 4 (CXCR4), which function as viral co-receptors.^7^

The interaction between the CCR5 receptor and its chemokines regulates the action of inflammatory cells.^8^ Chemokines condition the migration of leukocyte cells from blood to tissues during inflammatory responses, and dysregulation in this interaction or altered expression of chemokines and their receptors is correlated with different diseases.^9^

Innate and adaptive immunity are interdependent defense mechanisms of the immune system against invading pathogens, the innate immunity corresponding to the host's first line of defense that subsequently functions to coordinate the action of the adaptive response. As for innate immunity, it starts with the recognition of the pathogen through its pathogen-associated molecular patterns (PAMPs) that is done through pattern recognition receptors (PRRs), such as Toll-like receptors (TLRs, especially 7 and 8), retinoic acid-inducible gene I-like receptors—RIG-I (RLRs), melanoma differentiation-associated protein 5 (MDA-5), and cytosolic DNA sensors.^10^

In this sense, once HIV-1 PAMP is recognized by PRRs, a cascade of innate signaling pathways is generated, such as interferon regulatory factors (IRFs), inducing an antiviral response from effector responses of immune cell via inflammatory cytokines, IFNs, and caspase-1 activation pyroptosis.^11^ For the action of the innate defense mechanism, plasmacytoid dendritic cells (pDCs) play a central role in fighting the virus, even before its detectable circulation. In addition, monocytes and macrophages act in the recruitment of other immune cells and in the induction of mediators and in the stimulation of T cells in the long-lasting antigenic response. Furthermore, innate lymphoid cells (ILCs) and natural killer cells (NK), with their cytotoxic character, configure incisive antiviral roles after viremia.^12^

In adaptive defense mechanisms, T and B cell responses with specificity to HIV manifest weeks after plasma viremia, forcing successive mutations of the virus as a strategy to overcome these actions. T cells are associated with the differentiation and targeting of proinflammatory cytokines, relative to CD4^+^ cells, and the response through antiviral mechanisms over time. The importance of T cells in acute infection is characterized in the correlation between activation markers via memory CD4^+^ T cells (helper) present in the peripheral circulation with higher levels of CD4^+^ T cells at 2 years post-infection.^13^ CD4^+^ T lymphocytes are the primary cellular targets of HIV; however, the activation state of CD4^+^ T lymphocytes appears to have a significant impact on the virus' ability to infect these cells.^14^ With regard to the contribution of B T-bet^+^ cells, it has shown that these are linked with the memory response to the HIV-binding protein.^15^

The study by Zanoni et al demonstrated the significant action of CD8^+^ T cells (cytotoxic), with TCR (T cell receptor)-mediated activation, on HIV expression and replication through an immunoregulatory mechanism that prevents the proliferation and activation of infected CD4^+^ T cells, which are primary receptors for the virus.^16^ Infectious and non-infectious diseases can manifest in different ways in a population and can be influenced mainly by genetic components and environmental factors. As far as the genetic issue is concerned, when a gene undergoes a mutation and it reaches a percentage of presence equal to or greater than 1% in the population, it is considered a genetic polymorphism, whose classification is determined in its structure, distribution, stability, and form of transmission. In this sense, genetic polymorphisms consist of the genetic variability presented by a population of living beings.^17^ Although they are configured multivariately, in a particular way, this study will focus on single nucleotide polymorphisms (SNPs) and Insertions and Deletions (Indels).

Among the types of genetic polymorphisms, SNPs and Indels are most frequently found in humans, influencing both physiological changes and pathological repercussions for the patient.^18^ In this context, the association of susceptibility/protection with several diseases is described by distinct variations of SNPs and/or Indels, for example, in cytokine receptor or mediator genes, and in genes of these molecules, which have a highly polymorphic character, especially those involved in immune responses, as in HIV-1.^19^

At this juncture, an SNP consists of the exchange of a single nucleotide in regions of the gene, which may be located in non-coding regions (introns) or coding regions (exons).^20^ In the case of exons, due to their role in protein coding, SNPs can act in a synonymous way, when the nucleotide exchange does not generate amino acid change, or in a non-synonymous way, which can result in the premature end of transcription at the position of the mutated codon or the creation of a different amino acid from the standard one, altering the final protein whose function and efficiency may diverge from normal.^21^

^‡^The Indels, however, occur from insertions of new base pairs (bp) or deletions of native base pairs of a nucleotide sequence, which can compromise the stability of the DNA structure or, if they reach a coding region, alter the final protein product, as occurs with the Δ32 mutation in the human gene encoding the CCR5 protein, which, due to a deletion of 32 bp, results in a protein that is attached to the endoplasmic reticulum membrane and cannot lodge in the plasma membrane, which can make the individual resistant to HIV-1. This Indel modifies CCR5 surface expression and interfere with HIV-1 replication kinetics and thus the absence of a functional CCR5 at the cell surface as a coreceptor for the virus to enter the cell, the CCR5Δ32 mutation will reduce the risk of infection to this virus and is a key factor in HIV resistance.^22^

Over the past decades, genomic research on the host and HIV-1 has advanced, bringing candidate genes of immunogenic interest, due to advances in sequencing technologies and data science.^23^ Candidate genes are genes that provide biological effects on phenotypes (endophenotypes) based on functional variant sites, thus enabling the association between phenotypic manifestations of disease and the corresponding genes.^24^ With regard to HIV-1, one of the candidate genes is *CCR5*, due, among other nuances, to its function in cellular integration of the organism to the virus upon viral entry.

The *CCR5* gene has a length of 6,065 bases, is composed of 3 exons and 2 introns, and is located on the short arm of chromosome 3 in the p21.3 region (ID 1234). It is responsible for encoding the CCR5 protein, also known as the CCR5 receptor, a seven-domain transmembrane protein with three extracellular loops (ECL1, ECL2, and ECL3) and three intracellular loops (ICL1, ICL2, and ICL3).^25^ This receptor, like other chemokines, is present on several cells of the immune system, such as monocytes, immature dendritic cells, and lymphocytes.^26^

The CCR5 protein is mainly associated with immune system cell recruitment, proinflammatory activities, cell activation, and proliferation and is related to the response of the *T helper* (Th) 1 subpopulation of CD4^+^ T lymphocytes. Chemokine receptors act as a switch opening the target cell for HIV infection, CCR5 being the main co-receptor for HIV-1 strains with tropism for macrophages.^27^ Therefore, this protein is crucial for maintaining the efficiency of the human immune system by recruiting chemokines with effector, homeostatic, and inflammation-regulating functions.^25^

Cytokines are small molecules that promote functional cell signaling in the regulation and promotion of immune responses.^28^ A group of cytokines are called chemokines because they have chemostatic function.^29^ The interaction between chemokines that bind to the CCR5 receptor and this receptor regulates the action of inflammatory cells.^8^ Chemokines condition the migration of leukocyte cells from blood to tissues during inflammatory responses. Dysregulation in this interaction or altered expression of chemokines and their receptors is correlated with different diseases.^9^

The CCR5 receptor has a structure with seven α-helical transmembrane domains, coupled to the guanosine triphosphate-binding protein G (GTP). G proteins play a prominent role in mediating communication events through CCR5 and CXCR4 that are products for HIV infection. This receptor binds naturally to chemokines with an effector role in HIV-1, such as: macrophage inflammatory protein 1a (MIP-1a/CCL3); macrophage inflammatory protein 1b (MIP-1b/CCL4); regulated on activation, normal T cell expressed and secreted (RANTES/CCL5).^30^

Thus, genetic epidemiology was born to understand how these factors may be related through two main methodological approaches, which consist of linkage and association studies.^31^ Association studies consist of observing the allele frequencies in individuals from a population with the target disease, comparing it with healthy control groups belonging to the same population, and their main purpose is to find the role that different mutations of the same gene may play in the manifestation of a particular disease.^32^ In light of this, this review seeks to analyze the influence of genetic aspects (in *CCR5* polymorphisms) in association studies for HIV-1 infectious disease.

## Methods

### Study design

This is a systematic literature review, which aims to describe the correlations between the *CCR5* genetic polymorphisms and HIV published in the literature.

The study followed the formation stages: (1) Elaboration of the research question and problem; (2) Stipulation of inclusion and exclusion criteria; (3) Sampling; (4) Analysis of the articles; (5) Interpretation, discussion, and presentation of the review. The Preferred Reporting Items for Systematic Reviews and Meta-Analyses (PRISMA) flowchart, based on the PRISMA 2020 protocol, was used to present the steps followed for the present study.^33^

### Search strategy

The research problem was carried out, through which the following keywords were selected as a search strategy by an advanced search in the databases. First, we used the MeSH descriptors: “Receptors, CCR5”[Mesh]; “HIV”[Mesh]; “Polymorphism, Genetic”[Mesh], together with the Boolean operator “AND.” Then, another search was conducted with MeSH descriptors: “HIV”[Mesh]; “Receptors, CCR5”[Mesh]; “Polymorphism, Single Nucleotide”[Mesh], in conjunction with the Boolean Operator AND. The search took place in the following databases: U.S. National Library of Medicine National Institutes of Health (PubMed), Coordination for the Improvement of Higher Education Personnel (CAPES) Journal, and ScienceDirect.

### Eligibility criteria

The inclusion criteria outline articles written in English, Portuguese, or Spanish languages between 2001 and 2021 according to the population, exposure, comparator, outcome, and study design (PECOS), where:

(a) Population (P): patients infected with HIV;
(b) Exposure (E): involvement of *CCR5* genetic polymorphisms with HIV;
(c) Comparator (C): individuals with and without these genetic polymorphisms;
(d) Outcome (O): susceptibility or progression or higher transmission or gravity of HIV;
(e) Study design (S): Clinical trials, case–control studies, cross-sectional studies, or cohort studies published in scientific journals peer-reviewed.

Thus, the following question was raised: “Which *CCR5* genetic variants have proven susceptibility or protection and/or higher transmission and/or progression of HIV?”^34^ The justification for the time cut established was to analyze the scientific productions to verify the updates and evolution in the specific approach of the central theme of the study. Only Indel Delta32 was considered for inclusion in the criteria for this type of variant due to the large number of studies involving it.

Exclusion criteria were as follows: case reports, review articles, book chapters, theses, guidelines, letters to the editor, *in vitro* studies, and studies were available only the abstract and outside the stipulated period. Articles that did not evaluate the *CCR5* genetic polymorphisms through a molecular biology approach were also excluded. Articles that studied Indels other than Delta32 were also excluded.

### Study selection and review process

For the definition of *CCR5* variants studied here, we considered SNPs and Indel mutations. Excel software was used to organize and sort titles and abstracts. The data selection step for the search visualization was performed by two members (D.S.S. and M.J.A.S.) independently, thus ensuring their reliability. Any type of discord on the study selection and review process was resolved through discussion.

### Quality assessment

The Newcastle–Ottawa Scale (NOS) was used to assess the quality of the 32 included articles. A collaboration between two universities, the University of Newcastle in Australia and the University of Ottawa in Canada, produced this tool to assess the quality of nonrandomized studies, such as case–control and cohort studies with a score of 0–9. The NOS is divided into three domains: selection, comparability, and exposure/outcome.^35^ For cross-sectional studies, the NOS was cohort-adapted for these studies to promote reliable quality assessment (with a score of 0–10).^36^ The selection, comparability, and exposure stages of this scale assign values according to the specifics of each type of study, as shown below:

#### Case–control studies

The selection step is about the representativeness of the cases; type of controls selection; adequate controls definition. The comparability is about the comparability of cases and controls on the basis of the design or analysis. The exposure/outcome phase is divided into: ascertainment of exposure; same method of ascertainment for cases and controls; and nonresponse rate.

#### Cohort studies

The selection stage is about the representativeness of the exposed cohort; selection of the nonexposed cohort; ascertainment of exposure; demonstration that outcome of interest was not present at start of study. There is the comparability of cohorts on the basis of the design or analysis controlled for confounder; assessment of outcome; follow-up long enough for outcomes to occur; and adequacy of follow-up of cohorts.

#### Cross-sectional studies

The selection phase is about the representativeness of the sample; sample size; nonrespondents; ascertainment of the exposure (risk factor). The subjects in different outcome groups are comparable, based on the study design or analysis. Confounding factors are controlled; assessment of the outcome; and statistical test.

Therefore, four stars could be attribute to the selection, two stars(*) to the comparability, and three stars to the exposure (case–controls studies)/outcome (cohort studies). For the cross-sectional studies, five stars could be attributing to the selection, two stars to the comparability, and three stars to the outcome. This is a tool that scores the study on eight items. Six stars or more are classified as low risk of bias, and five stars or less as high risk of bias.^37^

### Data collection

A descriptive and tabular synthesis was carried out using the extracted data and major results of each included study. A team of two investigators extracted the data. The data included: (1) author information, (2) year of publishing, (3) information on the setting for each study (the genotyping method employed), (4) characteristics of study participants (phenotypic definitions and geographic characteristics), (5) characteristics of candidate genetic variation (type, locus, and evidence of functional role), and (6) outcome measure [genotypes and/or allele frequencies more associated and if the study had applied the Hardy–Weinberg equilibrium (HWE) test]. Significance degrees (*p* < .05) were considered statistically significant correlations between categories of each variation and this infectious disease studied.

## Results

**FIG. 1.**
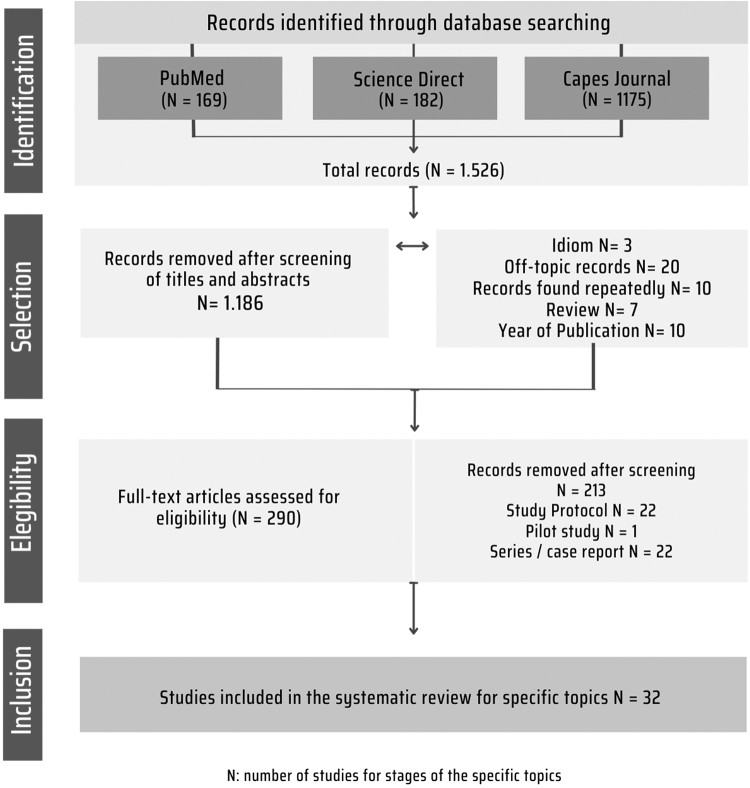
Flowchart depicting the study selection for systematic review. Belém (PA), Brazil, 2022.

We present the details of the search strategy in Figure 1. The numerical representation of the search for articles in the database in descending order was formed by CAPES Journal (*N* = 1,175), PubMed (*N* = 50), and ScienceDirect (*N* = 182). The journal with the highest number of publications on the investigated topic was the *AIDS Research and Human Retroviruses* (four studies). The final sampling (formed by 32 articles) is presented in Table 1.

**Table 1.** Characteristics of the Included Studies in This Review

No.	Author (year of publication)^Ref.^	Title	Database	Country	Method	Kind of study/participants	HWE testing (significant* p *value)	Mutation ID/type	Results	Newcastle–Ottawa Scale total score
(1)	Kageyama et al (2001)^38^	Polymorphism of *CCR5* affecting HIV disease progression in the Japanese population	PubMed	Japan	PCR genotyping	Cohort/98 HIV-1 patients	No	rs2856758 (SNP); rs2734648 (SNP); rs1799987 (SNP); rs1799988 (SNP); rs1800023 (SNP); rs1800024 (SNP)	The T mutant allele of SNP rs1799988 and the G wild allele of rs1800023 were associated with late onset of AIDS. In contrast, the A mutant allele of rs1800023 is associated with early onset of AIDS. This indicates that the cited SNP alleles are related to susceptibility and disease progression. For the other mutations, no associations were possible.	8^*^
(2)	Majumder and Dey (2001)^39^	Absence of the HIV-1 protective ΔCCR5 allele in most ethnic populations of India	PubMed/CAPES	India	PCR genotyping	Cohort/1,436 subjects	No	CCR5Δ32 (rs333)—Indel	^†^ **Correction:** This article has been updated on December 22, 2022 after first online publication of November 9, 2022 to clarify text on this page. ^†^This phrase formerly read: “There was no significant association for this Indel and the disease, as it is absent in most of the Indian ethnic groups investigated.” The protective allele of this Indel was rarely found in this investigated Indian population.	8^*^
(3)	Liu et al (2003)^40^	[Single nucleotide polymorphism loci of HIV-1 coreceptor *CCR5* gene in Chinese Han people]	PubMed	China	PCR genotyping	Cohort/725 subjects, including 287 HIV-1 SP patients, 388 HC ethnically matched for age, and 49 IDUs HESN	Yes (*p* > .05)	CCR5Δ32-rs333 (Indel); rs1800560 (SNP); rs1799987 (SNP)	For SNP rs1799987, the G mutant allele did not confer protection or susceptibility to HIV-1 and had a frequency of about 40% among the groups analyzed. For the other SNPs and Indel, no associations were possible.	8^*^
(4)	Salkowitz et al (2003)^41^	*CCR5* promoter polymorphism determines macrophage CCR5 density and magnitude of HIV-1 propagation *in vitro*	PubMed/ScienceDirect	United States	PCR genotyping	Cohort/18 healthy individuals to HIV-1	No	rs1799987 (SNP)	For SNP rs1799987, the A wild allele increases CCR5 expression (susceptibility) and predicts the magnitude of HIV-1 spread (severity).	7^*^
(5)	Singh et al (2003)^42^	Genetic influence of *CCR5*, *CCR2*, and *SDF1* variants on human immunodeficiency virus 1 (HIV-1)-related disease progression and neurological impairment, in children with symptomatic HIV-1 infection	PubMed	United States	PCR genotyping	Cohort/1,049 children with symptomatic HIV-1	No	CCR5Δ32-rs333 (Indel); rs1799987 (SNP); rs1799988 (SNP); rs184370729 (SNP)	For SNP rs1799987, the homozygous AA genotype showed a higher risk of disease progression. Indel Δ32 showed a protective role for the disease based on frequency in the cohort. For the other variants, no significant associations were possible.	9^*^
(6)	Duggal et al (2005)^43^	The effect of RANTES chemokine genetic variants on early HIV-1 plasma RNA among African American injection drug users	PubMed/CAPES	United States	Reverse transcription polymerase chain reaction (RT-PCR) and PCR-restriction fragment length polymorphism (RFLP) genotyping	Cohort/198 injecting drug users	No	CCR5Δ32-rs333 (Indel)	^‡^The protective allele of this Indel was rarely found in this investigated population. **Correction:** This article has been updated on December 22, 2022 after first online publication of November 9, 2022 to clarify text on this page. ^‡^This phrase formerly read: “There was no significant association between Indel and the disease.”	7^*^
(7)	de Souza et al (2006)^44^	*CCR5* promoter polymorphisms and HIV-1 perinatal transmission in Brazilian children	PubMed/CAPES	Brazil	PCR genotyping	Case–control/280 subjects, with 104 healthy children, 106 cases of children with the disease, and 70 children without HIV-1 (both born to HIV-1 positive women)	Yes (*p* > .05)	rs1799988 (SNP); rs184370729 (SNP); rs1800023 (SNP); rs1800024 (SNP); Δ32-rs333 (Indel)	For SNPs rs1799988 and rs1800023, the frequency differed among HIV+ children, HIV− children and HC. The presence of the TT genotype of 59353 indicated a trend toward increased risk of vertical transmission of HIV-1 infection, whereas the presence of the AA genotype of rs1800023 was suggestive of a protective effect against vertical transmission of the disease. For the other mutations, no significant associations were possible.	9^*^
(8)	Shrestha et al (2006)^45^	Behavioral risk exposure and host genetics of susceptibility to HIV-1 infection	PubMed/CAPES	United States	PCR genotyping	Paired case–control 1:2/291 patients and 532 controls	Yes (*p* > .05)	rs1799987 (SNP)	For SNP rs1799987, the wild-type A allele conferred HIV-1 susceptibility.	9^*^
(9)	Suresh et al (2006)^46^	Gene polymorphisms in *CCR5*, *CCR2*, *CX3CR1*, *SDF-1* and *RANTES* in exposed but uninfected partners of HIV-1 infected individuals in North India	PubMed/CAPES	India	PCR genotyping	Cohort/160 subjects (35 exposed but not HIV-1 infected, 75 HIV-1 seronegative normal HC, and 50 HIV-1-infected controls)	No	CCR5Δ32-rs333 (Indel)	There was no association between the CCR5-Δ32 polymorphism (rare mutation frequency among groups) and HIV-1 susceptibility in the cohort.	8^*^
(10)	Adojaan et al (2007)^47^	High prevalence of the CCR5Delta32 HIV-resistance mutation among Estonian HIV type 1-infected individuals	PubMed	Estonia	PCR genotyping	Cohort/303 subjects	No	CCR5Δ32-rs333 (Indel)	No difference was found between the frequencies of CCR5Δ32 deletion between IDUs and sexually infected persons.	8^*^
(11)	Kaur et al (2007)^48^	Polymorphism in the *CCR5* gene promoter and HIV-1 infection in North Indians	PubMed/CAPES/ScienceDirect	India	PCR genotyping	Cross-sectional study/180 patients and 119 controls	Yes (*p* > .05)	rs181651143 (SNP); rs1799988 (SNP); rs41469351 (SNP); rs1800023 (SNP); rs41469351 (SNP); CCR5Δ32-rs333 (Indel)	The A wild allele of SNP rs1800023 was more frequent in SP individuals, which characterizes a susceptibility risk effect. This allele was also found more in patients with stage C disease (more advanced) even compared with the two groups A and B together. The homozygous AA genotype was significantly elevated in SP individuals. For the other SNPs and Indel, there were no significant associations.	10^*^
(12)	Pedersen et al (2007)^49^	*CCR5* haplotypes and mother-to-child HIV transmission in Malawi	PubMed	Malawi	Real-time PCR-multiplex genotyping	Cohort/infants (*n* = 552) of HIV-positive women	Yes (uninformed)	rs2856758 (SNP); rs2734648 (SNP); rs1799987 (SNP); rs1799988 (SNP); rs41469351 (SNP); rs1800023 (SNP); rs1800024 (SNP); CCR5 Δ32-rs333 (Indel)	Overall, protection against MTCT was weakly associated with the two SNPs, the G mutant allele of rs1799987 and the T mutant of rs1799988. No children carried the Δ32 Indel. MVL was found to be an effect measure modifier. Among mothers with low MVL, statistically significant protection against MTCT was observed for the mutant alleles of the aforementioned mutations. Statistically significant protection was not found in high MVL. For the other SNPs, no significant associations were possible.	8^*^
(13)	Viladés et al (2007)^50^	Effect of genetic variants of *CCR2* and *CCL2* on the natural history of HIV-1 infection: CCL2-2518GG is overrepresented in a cohort of Spanish HIV-1-infected subjects	PubMed	Spain	PCR genotyping	Case–control/318 subjects, with 182 HIV-1-infected patients and 136 uninfected individuals (100 HC and 36 highly exposed but uninfected—EUs)	Yes (*p* > .05)	CCR5Δ32-rs333 (Indel)	There were no significant differences in the studied *CCR5* mutation genotype or allele distributions between the different groups. Homozygosity for the CCR5Δ32 mutant allele was not observed.	8^*^
(14)	Jang et al (2008)^51^	The effects of *RANTES/CCR5* promoter polymorphisms on HIV disease progression in HIV-infected Koreans	PubMed/CAPES	North Korea and South Korea	PCR-RFLP genotyping	Case–control/95 subjects (27 LTNPs, 29 patients, and 39 controls)	Yes (uninformed)	rs1799987 (SNP); rs1799988 (SNP); rs1800023 (SNP)	The C mutant allele of SNP rs1799988 was significantly more present in AIDS patients than in nonprogressive patients (LTNPs).	8^*^
(15)	Wichukchinda et al (2008)^52^	Effects of *CCR2* and *CCR5* polymorphisms on HIV-1 infection in Thai females	PubMed/CAPES	Thailand	PCR-RFLP genotyping	Case–control/421 subjects (74 exposed but uninfected women and 347 HIV-positive women)	No	rs1800452 (SNP)	No significant independent association between the cited SNP was possible.	8^*^
(16)	Parczewski et al (2009)^53^	Sequence variants of chemokine receptor genes and susceptibility to HIV-1 infection	PubMed	Poland	PCR-RFLP genotyping	Cohort/319 subjects (168 HIV-1-positive adult cases and 151 newborns—controls)	Yes (*p* > .05)	rs1799987 (SNP); CCR5Δ32-rs333 (Indel)	Both mutations were more frequent in the neonate group than in the SP group. For Indel Δ32, no homozygous Δ32/Δ32 genotype was found in the case group, but the mutant allele was found in the control group. This indicates a protective role of these polymorphisms for HIV-1.	9^*^
(17)	Rathore et al (2009)^54^	Association of *CCR5*-59029 A/G and *CCL3L1* copy number polymorphism with HIV type 1 transmission/progression among HIV type 1-seropositive and repeatedly sexually exposed HIV type 1-seronegative North Indians	PubMed	India	PCR and real-time TaqMan PCR genotyping	Cohort/558 subjects, divided into 196 patients, stratified into stages I, II, and III, plus 47 seronegative and 315 healthy volunteers	No	rs1799987 (SNP)	For SNP rs1799987, the G mutant allele was associated with disease protection. The heterozygous AG genotype of this SNP generated higher HIV-1 transmission.	8^*^
(18)	Veloso et al (2010)^55^	Effect of *TNF*-α genetic variants and CCR5Δ32 on the vulnerability to HIV-1 infection and disease progression in Caucasian Spaniards	PubMed/CAPES	Spain	PCR-RFLP genotyping	Cross-sectional study/423 subjects, comprising 239 uninfected (36 heavily exposed but not infected and 203 HC) and 184 infected (109 TP and 75 LTNPs of more than 16 years duration)	Yes (*p* > .05)	CCR5Δ32-rs333 (Indel)	The CCR5Δ32 distribution was not significantly different in HIV-1-infected patients compared with the uninfected population and between TP and LTNPs.	10^*^
(19)	van Manen et al (2011)^56^	Rising HIV-1 viral load set point at a population level coincides with a fading impact of host genetic factors on HIV-1 control	PubMed	Netherlands; Germany	PCR genotyping	Cohort/459 seroconverted individuals from pre-2003 cohorts, 335 German and 124 from the Netherlands, plus 231 seroconverted individuals from the post-2003 Netherlands cohort	Yes (uninformed)	CCR5Δ32-rs333 (Indel)	In post-2003 seroconverters, a higher viral load was found compared with pre-2003. The Δ32 mutant allele of Indel had a similar frequency in both groups and was individually associated with a lower viral load in pre-2003 seroconverters, although it was not independent of other genetic markers over time, assessed during the study.	9^*^
(20)	Xu et al (2011)^57^	A haplotype in the *CCR5* gene promoter was associated with the susceptibility to HIV-1 infection in a northern Chinese population	PubMed/CAPES	India	PCR genotyping	Case–control/148 subjects (78 SP and 70 controls)	Yes (*p* > .05)	rs2856758 (SNP); rs2734648 (SNP); rs1799987-59029 G/A (SNP); rs1799988-59353 T/C (SNP); rs1800023-59402 A/G (SNP); rs1800024-59653 C/T (SNP); CCR5Δ32-rs333 (Indel)	No mutant allele of CCR5Δ32 was observed in any of the individuals tested, regardless of HIV-1 infection status. The mutant allele at SNP rs2856758 had rare frequency. For all mutations, no significant associations were possible.	9^*^
(21)	Lu et al (2012)^58^	The genetic associations and epistatic effects of the *CCR5* promoter and CCR2-V64I polymorphisms on susceptibility to HIV-1 infection in a Northern Han Chinese population	PubMed	China	PCR genotyping	Case–control/182 subjects (91 patients and 91 controls)	Yes (*p* > .05)	rs2856758 (SNP); rs2734648 (SNP); rs1799987 (SNP); rs1799988 (SNP); rs1800023 (SNP); rs1800024 (SNP)	The G mutant allele of SNP rs2856758 was associated with a lower risk of HIV-1 infection, whereas the T mutant allele of SNP rs1800024 was significantly associated with susceptibility to infection. For the other SNPs, no significant associations were possible.	8^*^
(22)	Nkenfou et al (2013)^59^	Distribution of CCR5-Delta32, CCR5 promoter 59029 A/G, CCR2-64I and SDF1-3′A genetic polymorphisms in HIV-1 infected and uninfected patients in the west region of Cameroon	PubMed/CAPES	Cameroon	PCR-RFLP genotyping	Case–control/179 subjects (32 cases and 147 controls)	Yes (*p* > .05)	CCR5Δ32 (rs333)—Indel; rs1799987 (SNP)	No mutant alleles of Indel were found in the groups. For SNP rs1799987, the GG genotype was associated with a higher risk of developing HIV-1 infection.	8^*^
(23)	Zapata et al (2013)^60^	Influence of *CCR5* and *CCR2* genetic variants in the resistance/susceptibility to HIV in serodiscordant couples from Colombia	PubMed	Colombia	PCR genotyping	Cohort/239 subjects (70 HESN, 57 SP, and 112 HC)	Yes (*p* > .05)	CCR5Δ32-rs333 (Indel)	No subjects had homozygous Δ32 genotype, and few had heterozygous frequency, which did not generate significant associations for this mutation and the disease.	8^*^
(24)	Li et al (2014)^61^	Gene polymorphisms in *CCR5*, *CCR2*, *SDF1* and *RANTES* among Chinese Han population with HIV-1 infection	PubMed/ScienceDirect	China	PCR and PCR-RFLP genotyping	Cohort/724 subjects (287 HIV-seropositive and 388 controls and 49 IDUs)	Yes (*p* > .05)	CCR5D32-rs333 (Indel); rs1800560 (SNP); rs1799987 (SNP)	For the SNPs studied, the data showed that there were no significant associations for HIV-1.	9^*^
(25)	Gong et al (2015)^62^	A *SDF1* genetic variant confers resistance to HIV-1 infection in intravenous drug users in China	PubMed/CAPES/ScienceDirect	China	PCR genotyping	Cohort/921 male intravenous drug users (IDUs), divided into 263 HIV-1-exposed seropositive (HESP) and 658 HESN	Yes (*p* > .05)	rs2734225 (SNP); rs1799988 (SNP); rs1800452 (SNP); rs746492 (SNP)	For all the *CCR5* SNPs studied here, no correlations were possible.	8^*^
(26)	Gupta and Padh (2015)^63^	Analysis of *CCR5* and *SDF-1* genetic variants and HIV infection in Indian population	PubMed/CAPES	India	PCR and PCR-RFLP genotyping	Case–control/321 subjects (80 patients and 241 controls)	Yes (*p* < .05 for SNP 59029 and *p* > .05 for the other variants)	rs2734648 (SNP); rs1799987 (SNP); rs1799988 (SNP); rs41469351 (SNP); rs1800023 (SNP); rs1800024 (SNP); CCR5Δ32-rs333 (Indel)	For SNP rs41469351, the C wild allele was associated with increased risk of HIV-1 infection. Indel CCR5Δ32 was reported to be absent in the study population. The other SNPs had statistically insignificant frequencies.	8^*^
(27)	Mehlotra et al (2015)^64^	*CCR2*, *CCR5*, and *CXCL12* variation and HIV/AIDS in Papua New Guinea	PubMed/CAPES/ScienceDirect	Papua New Guinea; United States; Senegal; Guinea; Sierra Leone; Ivory Coast; Ghana	PCR genotyping	Cohort/(*n* = 258), North America (*n* = 184), and five West African countries (*n* = 178)	Yes (uninformed)	rs2856758 (SNP); rs1799987 (SNP); rs2734648 (SNP); rs1799988 (SNP); rs41469351 (SNP); rs1800023 (SNP); rs1800024 (SNP); CCR5Δ32-rs333 (Indel)	For SNP rs1799987, the wild-type A allele was associated with greater severity and susceptibility to HIV-1. For Indel Δ32, the presence of the mutation was associated with a protective role against HIV-1. For the other mutations, no significant associations were possible.	9^*^
(28)	Zifawei et al (2016)^65^	Null single nucleotide polymorphism in chemokine receptor 5 (*CCR5*) genes among the Ijaw ethnic population of Nigeria	PubMed	Nigeria	PCR genotyping and bioinformatics	Cross-sectional study/a total of 100 subjects were recruited for the study, among them, 75 (75%) were HIV-negative and 25 (25%) were HIV-positive controls	No	CCR5Δ32-rs333 (Indel)	The mutation was not found in any study individual. Therefore, no significant association was possible.	9^*^
(29)	Heydarifard et al (2017)^66^	Polymorphisms in CCR5Δ32 and risk of HIV-1 infection in the southeast of Caspian Sea, Iran	PubMed	Iran	PCR genotyping	Case–control/440 subjects (300 controls and 140 cases)	Yes (*p* < .05)	CCR5Δ32-rs333 (Indel)	The frequency of the Indel mutation was rare among the groups analyzed. Thus, a role in HIV-1 susceptibility was not observed.	8^*^
(30)	Ellwanger et al (2018)^25^	CCR5Δ32 in HCV infection, HCV/HIV co-infection, and HCV-related diseases	PubMed/CAPES	Brazil	PCR genotyping	Case–control/574 subjects, divided into 300 patients and 274 controls	Yes (*p* > .05)	CCR5Δ32-rs333 (Indel)	The deletion frequency was considered rare in the population. Furthermore, the genotype frequency was similar between the groups, with no statistically significant differences.	9^*^
(31)	Koor et al (2019)^67^	Cis-regulatory genetic variants in the *CCR5* gene and natural HIV-1 control in black South Africans	PubMed/ScienceDirect	South Africa	PCR genotyping	Cohort/145 subjects, 71 of whom were HIV-1 controllers (23 elite controllers, 37 CVs and 11 non-progressors with high viral load) and 74 progressors.	Yes (*p* > .05)	rs2856758 (SNP); rs2734648 (SNP); rs1799987 (SNP); rs1799988 (SNP); rs41469351 (SNP); rs1800023 (SNP); rs1800024 (SNP); rs746492 (SNP); CCR5Δ32-rs333 (Indel)	The A mutant allele of rs1799987 had lower frequency in the controllers and CVs versus progressors group. This lower occurrence was observed for the C mutant allele of rs1799988 and G wild allele of rs746492, respectively. For rs746492, the TG and GG genotypes were associated with HIV progression in all the groups analyzed. However, among progressors there was a higher frequency of heterozygosity. Possession of the GG genotype of SNP rs746492 was associated with HIV-1 progression. For the other SNPs and Indel, no significant associations were possible.	7^*^
(32)	Donyavi et al (2020)^68^	Evaluation of CCR5-Δ32 mutation among individuals with high risk behaviors, neonates born to HIV-1 infected mothers, HIV-1 infected individuals, and healthy people in an Iranian population	PubMed/CAPES	Iran	PCR genotyping	Cross-sectional study/371 subjects, divided into 140 healthy Iranian people, 84 newborns of HIV-1-infected mothers, 71 people with high-risk behaviors, and 76 HIV-1-infected individual	No	CCR5Δ32 (rs333)—Indel	No significant associations were observed.	8^*^

AIDS, acquired immune deficiency syndrome; CAPES, Coordination for the Improvement of Higher Education Personnel; CCR5, C-C chemokine receptor 5; HC, healthy controls; HESN, HIV-1-seronegative individuals exposed to HIV-1; HIV, human immunodeficiency virus; HWE, Hardy–Weinberg equilibrium; IDU, intravenous drug user; Indel, Insertion and Deletion; LTNP, long-term non-progressor; MTCT, Mother-to-child transmission; MVL, maternal viral load; PCR, polymerase chain reaction; RANTES, regulated on activation, normal T cell expressed and secreted; SNP, single nucleotide polymorphism; SP, seropositive; TNF-α, tumor necrosis factor alpha; TP, typical progressors; VC, viremic controller.

**Correction:** This article has been updated on December 22, 2022 after first online publication of November 9, 2022 to clarify text on this page. ^†^This phrase formerly read: “There was no significant association for this Indel and the disease, as it is absent in most of the Indian ethnic groups investigated.”

**Correction:** This article has been updated on December 22, 2022 after first online publication of November 9, 2022 to clarify text on this page. ^‡^This phrase formerly read: “There was no significant association between Indel and the disease.”

## Discussion

The pathophysiology of HIV infection involves cellular interaction factors that utilize the host organism for adsorption, penetration, for reverse transcription to DNA, entry into the nucleus, integration with host DNA, transcription of viral RNA, assembly, and release.^69^ In this sense, scientific evidence states that among the cellular and molecular aspects that are involved in this context, mechanisms that promote virus replication, such as the expression of CCR5, CXCR4, CD4, and CypA, as well as influences contrary to viral replication represented by TRIM5α and APOBEC3G, gain the leading role.^70^ In particular, one sees immunogenetic interest in addressing the CCR5 receptor, which is encoded by the gene itself, the key point of this study.

C-C type chemokine receptors (CCRs) are given a subclass segmentation based on the chemokines they can recognize.^71^ CCR5 is found on T lymphocytes, B cells, microglia, and cells of the monocyte/macrophage lineage. The expression of CCR5 on T cell surfaces designates susceptibility to HIV/AIDS through modification in its activation. This parameter is determined by DNA methylation in *CCR5* cis-regulatory regions or cis-regions. With respect to cis-regions of *CCR5*, DNA methylation of dinucleotide cytosine–guanine (CpG) regions can elevate T cell methylation, producing higher levels of *CCR5* expression, as can its inverse relationship.^72^

CCR5 is the major chemokine receptor linked to HIV-1 transmission and progression, and the most potent chemokine as a natural ligand is called MIP-1alpha, which is also induced by this same receptor.^54^ Regarding HIV-1 infection initiation mechanisms, the beginning of the adsorption phase involves the binding of virions to the surface of target cells. This is mediated by a high-affinity interaction between the extracellular domain of the viral glycoprotein gp120 and specific cellular receptors, with the CD4^+^ T lymphocyte being the main receptor.^6^

After binding of this glycoprotein to the CD4^+^ T receptor, structural changes occur in the cell membrane that enable the interaction with co-receptors, such as CCR5, which allows fusion of the viral envelope with the host cell membrane and thus enables viral entry.^73^ On HIV-1 progression, viral load (replication) is inversely correlated with the quantity and quality of CD4^+^ T lymphocytes, which would be naturally responsible for releasing mediators from Th1, Th2 subpopulations, regulatory T cell (Tregs), Th17, and Th22 to deal with the virus. In addition, immune cells such as CD4^+^ T lymphocytes with reduced levels of *CCR5* would be more resistant to infection.^74^ Furthermore, CD8^+^ T cells can block cell-to-cell spread of HIV-1 through CCR5-binding chemokines (i.e., MIP-1a/CCL3, MIP-1b/CCL4, and RANTES/CCL5), as these competitively inhibit HIV entry via CCR5 expression and can prevent replication and pathogenesis.^16^

The host's genetic susceptibility to infection, transmission, and severity of disease is variable between individuals and populations. This may be driven by genetic, epigenetic, immunological, and/or environmental factors. Thus, even though studies report genetic associations for diseases, it was noted in the results of these surveys that there may be divergent data on outcomes derived from cases in different populations. From this point of view, some authors consider that this fact is due, above all, to the *genetic background*, which determines variability through differentiated genetic identity among individuals.^54,75,76^

The target gene can be studied for possible therapeutic applications in HIV-1 patients by various techniques, such as gene therapy for immunomodulation. A good example within this possibility of therapy is contained in the T-peptide, an amino acid molecule homologous to a binding epitope of gp120 (protein linked to adsorption on the host cell next to *CCR5*), wild-type or mutant alleles of genetic variants for patients (such as for Indel Δ32), or inhibition of viral infection by anti-CCR5 ribozymes used in the transplantation of immune cells from the bone marrow to the patient.^77–80^

Moreover, other HIV-1 treatment genetic variability includes CCR5 antagonist drugs [such as Maraviroc (MVC)] that have a direct positive impact on antiretroviral treatment,^81^ cytokine antagonist drugs, such as against tumor necrosis factor alpha (TNF-alpha), produced by Th1 lymphocyte subpopulation (which are also stimulated by proteins, such as CCR5),^82^ molecular therapies using CRISPR-Cas9 for gene editing in regions such as *CCR5*,^83^ generation of monoclonal antibodies to inhibit virus tropism to CCR5,^84^ and formation of predictive measures of severity by genetic counseling for polymorphic variants of *CCR5*.^85^

Of the 32 publications analyzed in this research, the major number of articles included was cohort studies (17 researches, 53.12%). The geographical relationship of production showed that most studies came from the Asian continent (15 researches, with 46.88%), but from countries other than Asia, especially China (with a contribution of 12.5% in total—4 researches). However, the direction of the populations studied in the entirety of the studies had a greater focus on American populations (15.63%), equivalent to five of them.

The findings of this research expose a variability of genetic aspects that relate polymorphisms associated with HIV-1 infection. Regarding the perspective of susceptibility or protection of the human organism against HIV-1 infection, the SNPs and Indels mentioned in the articles were as follows: rs2856758, rs1800024, rs1800023, rs1799987, Δ32 (rs333), rs1799988, rs746492. In a restricted manner, it is valid to point out that, with regard to the susceptibility aspect concerning polymorphisms associated with the disease in question, only the SNPs showed this correlation.

In this sense, the SNPs that had this associative character were as follows: T mutant allele of rs1800024 in Chinese population;^58^ C mutant allele of rs1799988 in North and South Korean populations;^51^ the A wild allele of rs1800023 in Indian population;^48^ the A wild allele of rs1799987 in the U.S. population;^45^ the A mutant allele of rs1800023 in Japanese population;^38^ C wild allele of rs41469351 in Indian population;^63^ the A wild allele of rs1799987 in Papua New Guinea, the United States, Senegal, Guinea, Sierra Leone, Ivory Coast, and Ghana populations; the A wild allele of rs1799987 in the U.S. population;^64^ the GG genotype of rs1799987 in Cameroon population.^59^ The SNP most often associated with susceptibility was rs1799987, present in four studies (12.5%), under the presence of the wild-type A allele.

However, the polymorphisms that had an associative character with HIV-1 protection were the SNPs: G mutant allele of rs2856758 in Chinese population;^58^ G mutant allele of rs1799987 in Polish population;^53^ G mutant allele of rs1799987 in Indian population.^54^ The SNP most associated with protection was also rs1799987, in two of the three studies of resistance conferred to infection (75%), under presence of the G mutant allele. Indel Δ32 exclusively guaranteed associations regarding protection, found by only three studies (12.5%) to confer resistance to HIV-1. In this context, this Indel obtained these mentions in the U.S. population;^42^ in a Polish population;^53^ in populations from Papua New Guinea, the United States, Senegal, Guinea, Sierra Leone, Ivory Coast, and Ghana.^64^ Fourteen studies were found with no association between the Indel cited and infection. Of these, 4 studies (23.53%) did not even find the deletion allele because of its rarity, and in the remaining studies (10 studies, 58.82%), their frequency measurements did not allow correlations.

Regarding the severity or delay of the disease, the correlated genetic polymorphisms were as follows: rs746492, rs1799987, rs1799988, Δ32, rs1800023.

Thus, the severity of the disease is designated by the following polymorphisms: the A wild allele of rs1799987, C mutant allele of rs1799988, and G mutant allele of rs746492 in the South African population.^67^ In a cohort study by van Manen et al, the presence of Δ32 in a group of seroconverters post-2003 was related to a higher viral load (progression) compared with pre-2003 in the German and Netherlands population;^56^ C mutant allele of rs1799988 in North and South Korean population;^51^ the A wild allele of rs1800023 in Indian population;^48^ the A wild allele of rs1799987 in the U.S. population;^42^ the A mutant allele of rs1800023 in Japanese population;^38^ the A wild allele of rs1799987 in populations from Papua New Guinea, the United States, Senegal, Guinea, Sierra Leone, Ivory Coast, and Ghana;^64^ the A wild allele of rs1799987 in the U.S. population.^41^

Regarding the delay of HIV-1-generated disease, only one study has identified two SNPs, the T allele of rs1799988 and the G wild allele of rs1800023 in a Japanese population.^38^

With regard to disease transmission, associations were possible for the SNPs: rs1799987, rs1799988, rs1800023. As far as the type of vertical transmission is concerned, the associative presentations of polymorphisms were established by: G mutant allele of rs1799987 and T mutant allele of rs1799988 conferred higher maternal–infant transmission in Malawi population;^49^ the TT genotype of rs1799988 generated higher vertical transmission, whereas the AA genotype of rs1800023 is suggestive of protective effect against vertical transmission of HIV-1 in Brazilian population.^44^ Thus, it can be seen that in two of three studies on transmission for the disease, the SNP rs1799987 provided greater transmissibility to the virus.

As for sexual transmission or transmission by contaminated blood products, only one study showed a positive correlation, which was designated by the AG genotype of rs1799987 that provided greater transmission of the virus between individuals in Indian population.^54^ Specifically, maternal–infant gestational transmission in the face of distinct HIV-1 genetic subtypes may generate variable transmission rates, thus being an important factor to consider for the genetic evaluation of *CCR5* mutations and a weak point of this review.^86^ Moreover, the elements that guide the rate of sexual transmission of HIV-1 between individuals and through contaminated blood fluids are also related to progression (high viremia), immunological aspects (immunocompromise) and other aspects, such as the presence of sexually transmitted infections (STIs) that make it difficult to assertively analyze genetic influences in the prevention process.^87^

It is noteworthy, however, that the following SNPs and Indels were not amenable to associations regarding the investigated questions of susceptibility, severity, and transmissibility in this review: Δ32 in Brazilian population;^25^ Δ32, rs1800024, rs1800023, rs41469351, rs2856758, rs2734648 in South African population;^67^ Δ32, rs1800560, rs1799987 in Chinese population;^61^ rs2734648, rs1799987, rs1799988, rs1800023 in Chinese population;^58^ Δ32 in Spanish population;^55^ rs2856758, rs2734648, rs1799987, rs1799988, rs1800023, rs1800024, Δ32 in Indian population;^57^ rs1799987, rs1800023 in North and South Korean population;^51^ rs1800452 in Thai population;^52^ Δ32, rs41469351, rs1799987, rs1799988, rs41469351 in Indian population;^48^ Δ32 in population of Estonia;^47^ Δ32 in another Spanish population;^50^ rs1799988, rs184370729 in the U.S. population;^42^ rs1800560, Δ32 in Chinese population.^40^

Furthermore, no statistical significance could be observed for SNPs and Indels in this review for: rs1800024, rs2856758, rs2734648, rs1799987 in Japanese population;^38^ rs2734648, rs1799987, rs1799988, rs1800023, rs1800024 in Indian population;^63^ Δ32 in Colombian population;^60^ Δ32 in Nigerian population;^65^ Δ32 in Iranian population;^66^ rs2856758, rs2734648, rs41469351, rs1800023, rs1800024 in Malawi population;^49^ Δ32 in another Indian population;^46^ Δ32 in another U.S. population;^43^ rs2856758, rs2734648, rs1799988, rs41469351, rs1800023, rs1800024 in populations of Papua New Guinea, the United States, Senegal, Guinea, Sierra Leone, Ivory Coast, and Ghana;^64^ Δ32 in another Indian population;^39^ rs184370729, rs1800024, Δ32 in Brazilian population;^44^ Δ32 in Cameroon population;^59^ rs2734225, rs1799988, rs1800452, rs746492 in Chinese population;^62^ Δ32 in another Iranian population.^68^

The numerical offering of findings involving *CCR5* gene polymorphisms expose majority targeting of Indel Δ32 (rs333), equivalent to 75% approaches in the total number of selected studies. Furthermore, the presentation of the most studied SNPs in descending order was rs1799987 (53.13%, in 17 studies), rs1799988 (with 12 studies, 37.5%), and rs1800023 (with 10 studies, 31.25%). It is worth mentioning that the SNP rs1799987 obtained a significant numerical role (6 studies, 18.75%) in the preponderant susceptibility, severity, and transmissibility of HIV-1 infection. Thus, there was a need to present the effects of the aforementioned variants that were most prominent in *CCR5* gene expression.

Indel Δ32 (rs333) is a 32 bp deletion in the featured gene, of the frameshift type in the coding region (exon 3). A frameshift mutation is an insertion or deletion of a nucleotide, in which the deleted number of base pairs is not divisible by 3.^88^ This interferes with the cell reading the DNA sequence of the gene by virtue of the fact that the cell can only read the triplet frame. Consequently, a stop codon is introduced early in the gene and produces a defective receptor.

The mutation causes amino acid substitution from serine (Ser) to isoleucine (Ile) and affects the ECL2, ICL3, ECL3, and the N-terminal regions of the protein. It is considered the most studied mutation in HIV-1 immunogenetics because it has been associated with relative disease resistance since 1996.^85^ The outcome of the presence of this mutation in the phenotypic manifestation of the disease is the control of the inflammatory response since this control is related to the progression of autoimmune and infectious diseases.^89^ CCR5 Δ32 to date is the only genetic mutation that completely blocks HIV-1 infection in humans.^70^

The rs1799987 also known as position 1 (p1), -2459G/A or 50929A is an SNP in intron 2 in the downstream promoter (Pd) of the gene, so it does not cause an amino acid change.^90^ It has been reported in studies that people with the A wild allele of this SNP expressed more of the gene and thus the CCR5 protein on the cell surfaces of CD4^+^ T cells, leading to severe HIV pathogenesis, because the activity at the promoter sites controls the death and apoptosis of CD4^+^ T cells.^91^ This promoter region is responsible for most of the transcriptional activity in active cells. Since the activity of this promoter is associated with mutation and positive regulation of CCR5; therefore, it increases the messenger RNA (mRNA) production levels of the virus. With respect to the protective aspect of this SNP against viral disease, the G mutant allele may become useful for developing antibodies against CCR5, producing neutralizing antibodies to HIV-2, and designing therapeutic vaccines for HIV-1.^91^

The rs1799988 also called 2135C/T or 59353T is an SNP in exon 2 of the gene, with an amino acid unknown change [in the 5′ untranslated region (5′UTR)]. The presence of this mutation influences in an unclear way molecularly the expression of this gene but reduces the expression of CCR5 on the surface of peripheral blood mononuclear cells, CD4^+^ cells, and CD4^+^ monocytes, contributing in this disease to lower acquisition and progression,^92^ as was shown by the studies found in this review. The rs1800023 also known as -2086A/G or 59402G is an SNP located in exon 2 of the gene (with unknown amino acid modification)—in the 5′UTR region, at a site from which most transcripts originate. The question of functional alteration in the gene remains unclear, although it has been determined that it promotes local inflammatory responses and thus may decrease the pathogenesis of diseases.^93^

In the systematic review by Reiche et al, the analyzed parameters contained the time cut from 1988 to 2006 on several polymorphisms in various genes, among them the *CCR5* gene, showing Indel Δ32 with a conference of protection against transmission in only one study, resistance to disease progression in nine, and defense against susceptibility in one, besides having no association in two studies. Of this amount, only 4 of the 13 CCR5 searches (30.77%) were involved in the period from 2001 onward (one of them with no association for this Indel and the disease).^85^

The meta-analysis by Liu et al presented a targeted approach to Indel Δ*32* upon selection of 18 studies with temporality between 1996 and July 2011, indicating parameters not associated with HIV-1 susceptibility.^94^ As for the meta-analysis by Ni et al, it also evaluated only Indel Δ32 from 24 case–control studies selected without language restriction in the final sample, in the temporal cut from 1996 to 2018, presenting results that this mutation may confer a possible protective role against HIV-1, both to susceptibility and disease severity.^95^ Thus, the data configuration can be defined with contrasting aspects between the aforementioned reviews from their population approaches and selection methods.

Therefore, this is the first systematic research that uses the synergistic analysis of both SNPs and Indel Δ32 regarding the parameter of polymorphisms that relate the *CCR5* gene to HIV-1 infection in terms of susceptibility, severity, and transmissibility, presenting a temporal counterpoint of the aforementioned studies before the approach in temporality of 20 years. Furthermore, this study is innovative in that it expressively presents the numerical significance of SNPs, as well as their variability and effects. Previous reviews on SNPs have also framed Indel Δ32 by virtue of its clinical importance in HIV-1 outcome and bring results that corroborate this present review.

The review analyses of this research have highlighted SNPs in the promoter region (Pd) of the *CCR5* gene, which have been shown in some populations to play associative roles in HIV-1 susceptibility, progression, and/or transmission. The domain analysis between the mutated sequence in the resulting Pd promoter region and similar consensus factor sequences indicated importance in most of them in *CCR5* and, as a consequence, factors responsible for connectivity between this region may be responsible for the heterogeneity of expression in this gene.^96^ This conjuncture is associated in several researches, mainly, emphasizing the A wild allele of rs1799987, which preponderantly provides susceptibility and disease progression characters.^70,85,97^

The *CCR5* gene corresponds downstream of *CCR2*, so the two are very close genes and this may imply a relationship between their polymorphisms.^90^ In addition, there is a long non-coding RNA (lncRNA) gene overlapping *CCR5* (called *CCR5AS*), whose expression is positively correlated with CCR5 mRNA levels.^98^ In this logic, polymorphic variants in *CCR2* and in *CCR5AS* are also determinant for association analyses with this disease from CCR5 levels in host cells.

The limitations of the study come up against: (1) each study's respective definition of HIV-1 infection based on case identification; (2) the SNPs used for evaluation in this review satisfy the requirement of being referenced in the National Center for Biotechnology Information (NCBI); (3) for the inclusion of studies, research with the *CCR5AS* gene, the antisense RNA form of *CCR5* that has great repercussion on *CCR5* gene expression were not considered because they are different genes and generate different polymorphisms; (4) heterogeneity of SNPs acting as a possible bias in characteristics such as ethnicities and ages of different populations due to the genetic background phenomenon; (5) regarding mother-to-child transmission, besides the *CCR5* gene variants, the different genetic subtypes of HIV-1 may also contribute to this process; (6) the need for joint analysis of mutations in *CCR2* and *CCR5* genes because of their structural proximity; (7) the need for joint analysis of polymorphic variants of *CCR5* and *CXCR4* because both are co-receptors for HIV-1, individually or jointly, responsible for viral entry into the host cell; (8) the need for data evaluation in studies of different variants of HIV-1; (9) possible evaluations in other types of *CCR5* genetic polymorphisms; (10) framing only one Indel (the Δ32) in this review because it is the most studied for HIV-1 infection, thus causing a possible selection bias of the polymorphisms represented here.

Therefore, genetic aspects influence HIV-1 infection in terms of susceptibility variables, transmission, and progression to AIDS. In this perspective, new genomic association studies with approaches in cohort studies, case–control studies, cross-sectional studies with larger sample or population sizes, and with populations from different countries or localities not yet analyzed may make it clearer to what extent immunogenetics may determine in the manifestation and establishment of HIV-1, as well as decipher the functional molecular mechanisms of virus propagation in cells.

## Conclusions

This review provides information engendering the polymorphic variants of *CCR5* as a potential gene of interest for association with disease. Polymorphic *CCR5* variants may decisively influence genetic aspects of HIV-1 infection through changes in transcriptional signaling, downregulation of gene expression, and overactivation of the inflammatory process. This study exposes that ^†^SNP rs1799987 is one of the genetic polymorphisms most associated with the criteria of susceptibility and severity of HIV-1, having distinct consequences in genotypic, allelic, and clinical analysis in the variability of investigated populations. As for the transmission character of the disease, the G mutant allele of rs1799987 corresponds to the highest positive association in cases of maternal–infant transmission, sexual transmission, and contact with sharps of the virus. ^‡^Furthermore, the results on Indel Δ32 corroborate the absence and rarity of this variant in some populations.

As for mitigating the severity of cases, and therefore delaying the disease, the SNPs rs1799988 and rs1800023 obtained significant attribution in individuals from the populations studied. Such data about the polymorphisms mentioned above are relevant for modulating the dynamics of replication, propagation, viremia, and vertical/sexual transmission based on distinct expressions of the *CCR5* gene, in addition to highlighting mutations of immunogenetic interest that can be used for future research involving approaches that include other populations and larger sample sizes. Therefore, the analysis of genetic studies on HIV-1 may provide possible innovative directions in diagnostic, predictive, therapeutic, and prophylactic measures. Such information is essential in the formulation of therapeutic proposals aimed at blocking the establishment of HIV-1, helping to corroborate the confrontation of the disease through new approaches.

## Data Sharing Statement

Data availability is not applicable, as it is a literature review.

## Notes

**Correction:** This article has been updated on December 22, 2022 after first online publication of November 9, 2022 to clarify text on this page. ^†^This phrase formerly read: “different recombinant forms: A, B, C, D, F, G, H, J, and K”.

**Correction:** This article has been updated on December 22, 2022 after first online publication of November 9, 2022 to clarify text on this page. ^‡^This paragraph formerly read: “The Indels, however, occur from insertions of new base pairs (bp) or deletions of native base pairs of a nucleotide sequence, which can compromise the stability of the DNA structure or, if they reach a coding region, alter the final protein product,^22^ as occurs with the Δ32 mutation in the human gene encoding the CCR5 protein, which, due to a deletion of 32 bp, originates a mutant receptor called CCR5Δ32, which can make the individual resistant to HIV-1.^22^”

**Correction:** This article has been updated on December 22, 2022 after first online publication of November 9, 2022 to clarify text on this page: ^†^This phrase formerly read: “The SNP rs1799987 is the genetic polymorphism most associated with HIV-1 susceptibility and severity criteria,”. ^‡^This sentence formerly read: “Furthermore, the results about the generic role of Indel Δ32 in this study corroborate the non-association of this variant with the protective function in HIV-1 infection.”
